# Risk factors for Post-PCI cardiovascular events in coronary artery disease patients treated with clopidogrel combined with aspirin

**DOI:** 10.3389/fphar.2026.1809310

**Published:** 2026-06-18

**Authors:** Jun Zhou, Qiang-Sheng Wang

**Affiliations:** 1 Department of Cardiology, Tongren Hospital, Shanghai Jiao Tong University School of Medicine, Shanghai, China; 2 Department of General Practice, The Headquarters of Beijing Nuclear Industry Hospital, Beijing, China

**Keywords:** aspirin, clopidogrel, coronary artery disease, dual antiplatelet therapy, major adverse cardiovascular events, percutaneous coronary intervention

## Abstract

**Background:**

Cytochrome P450 family two subfamily C member 19 (CYP2C19) loss-of-function (LOF) variants may influence clopidogrel response after percutaneous coronary intervention (PCI), but their prognostic relevance within combined clinical and procedural risk assessment remains incompletely defined. This study aimed to evaluate the association between CYP2C19 functional phenotype and major adverse cardiac and cerebrovascular events (MACCE) in coronary artery disease (CAD) patients receiving clopidogrel-based dual antiplatelet therapy (DAPT) after PCI.

**Method:**

This retrospective observational study included 280 consecutive CAD patients who underwent PCI with stent implantation and received aspirin plus clopidogrel. The primary endpoint was time to first MACCE. Multivariable Cox proportional hazards regression was the primary analysis, with logistic regression as a secondary supportive analysis. Kaplan-Meier analysis, subgroup interaction analyses, and sensitivity analyses were performed.

**Results:**

During a median follow-up of 32.0 months, 52 patients experienced MACCE (18.6%). In the primary Cox model, older age, diabetes mellitus, lower estimated glomerular filtration rate, lower left ventricular ejection fraction, greater total stent length, post-procedural Thrombolysis In Myocardial Infarction (MI) flow <3, and CYP2C19 LOF phenotype were associated with time to first MACCE. CYP2C19 LOF remained significant after adjustment (adjusted hazard ratio 1.74, 95% confidence interval 1.10–2.75; P = 0.018). Kaplan-Meier analysis showed lower MACCE-free survival in LOF carriers than in non-LOF patients (log-rank P = 0.007). Interaction analyses suggested stronger LOF-associated risk patterns in acute coronary syndrome and complex PCI subgroups.

**Conclusion:**

In clopidogrel-treated CAD patients after PCI, CYP2C19 LOF phenotype was associated with MACCE in a clinical–procedural risk framework. These findings support further prospective validation of integrated genotype-informed post-PCI risk stratification.

## 1 Introduction

Percutaneous coronary intervention (PCI) with stent implantation is a cornerstone treatment for patients with coronary artery disease (CAD), including those with stable CAD and acute coronary syndrome (ACS) (Virani et al., 2023; Vrints et al., 2024). Dual antiplatelet therapy (DAPT) with aspirin and a P2Y12 inhibitor is routinely used after PCI to reduce recurrent ischemic events (Byrne et al., 2023). Clopidogrel remains widely prescribed in clinical practice because of its established efficacy, safety profile, affordability, and broad availability (Wang et al., 2025; Kereiakes, 2025). However, major adverse cardiac and cerebrovascular events (MACCE), including cardiac death, non-fatal myocardial infarction (MI), ischemic stroke, and target vessel revascularization (TVR), continue to occur in a subset of post-PCI patients despite clopidogrel-based DAPT (Yu et al., 2024; Ayaz et al., 2025).

Post-PCI ischemic risk is multifactorial and reflects the combined influence of patient-level clinical characteristics, cardiovascular comorbidities, procedural complexity, coronary flow restoration, treatment adherence, and pharmacogenetic variability (Mohammed et al., 2025; Patel et al., 2025). Older age, diabetes mellitus, renal dysfunction, reduced left ventricular ejection fraction, extensive stent implantation, and impaired final Thrombolysis In MI flow have all been associated with adverse outcomes after PCI (Dello Russo et al., 2026). In parallel, Cytochrome P450 family two subfamily C member 19 (CYP2C19) loss-of-function (LOF) alleles may reduce clopidogrel bioactivation and attenuate antiplatelet response, whereas the clinical relevance of other candidate genes, such as ABCB1 and PON1, remains less consistent. Therefore, evaluation of post-PCI risk may require an integrated framework that considers both conventional clinical-procedural factors and CYP2C19 functional phenotype (Deng et al., 2024).

Although the association between CYP2C19 LOF alleles and reduced clopidogrel responsiveness after PCI has been well described, an important gap remains in how this genetic information should be interpreted within the broader clinical context of post-PCI risk (Mohammed et al., 2025; Biswas et al., 2024). Previous studies have primarily focused on the prognostic effect of CYP2C19 genotype itself, whereas less attention has been paid to whether CYP2C19 LOF status retains prognostic relevance when evaluated together with baseline clinical vulnerability and procedural complexity in a real-world clopidogrel-treated PCI population (Wang et al., 2021; Kim et al., 2024). In particular, limited evidence is available regarding whether the association between CYP2C19 LOF phenotype and adverse cardiovascular outcomes differs across clinically important subgroups, such as patients presenting with ACS or undergoing complex PCI(Park et al., 2026). To address this gap, the present study evaluated the association of CYP2C19 functional phenotype with time to first MACCE among CAD patients receiving clopidogrel plus aspirin after PCI, while integrating clinical factors, procedural characteristics, and prespecified subgroup interaction analyses.

## 2 Methods

### 2.1 Study design

This retrospective observational study enrolled consecutive patients with confirmed CAD who underwent PCI and received post-procedural DAPT with clopidogrel combined with aspirin at our institution between June 2020 and June 2025. Eligible participants were adults with angiographically documented CAD who completed PCI with stent implantation, had initiation of clopidogrel plus aspirin within 24 h after the index procedure, and had sufficient clinical records to ascertain baseline characteristics, procedural details, concomitant medications, and follow-up cardiovascular outcomes. Patients were excluded if they (1) lacked key exposure or outcome data; (2) did not receive the specified clopidogrel–aspirin regimen after PCI or discontinued dual therapy immediately for nonclinical reasons; (3) required long-term oral anticoagulation at discharge (for example, atrial fibrillation) or had other indications necessitating alternative antithrombotic strategies; (4) had active bleeding, a history of intracranial hemorrhage, severe thrombocytopenia, or known hypersensitivity/contraindications to aspirin or clopidogrel; (5) underwent concomitant cardiac surgery during the index hospitalization; or (6) had severe comorbid conditions expected to markedly limit survival or preclude outcome assessment during follow-up. Informed consent was obtained from all participants and/or their legal guardians. The study was approved by the ethics committee of our hospital and conducted in accordance with relevant guidelines and the Declaration of Helsinki. Data were anonymized prior to analysis to ensure confidentiality and protect participant privacy.

### 2.2 Treatment protocol

Post-PCI DAPT was defined as concomitant use of aspirin and clopidogrel. Aspirin was prescribed as a loading dose of 300 mg followed by a maintenance dose of 100 mg once daily. Clopidogrel was prescribed as a loading dose of 300–600 mg followed by a maintenance dose of 75 mg once daily. DAPT initiation was defined as receipt of both agents within 24 h after the index PCI, as documented in medication administration records and discharge prescriptions. The planned DAPT duration was extracted from discharge orders, and the actual duration was determined using outpatient follow-up records and prescription documentation, including dates of discontinuation or switching. Concomitant secondary prevention therapies were recorded at discharge and during follow-up, including statins, β-blockers, ACE inhibitors/angiotensin receptor blockers, angiotensin receptor–neprilysin inhibitors, and proton pump inhibitors. DAPT exposure stability was assessed using inpatient medication administration records, discharge prescriptions, outpatient prescriptions, and follow-up documentation. Actual DAPT duration was defined as the time from the index PCI to discontinuation of either aspirin or clopidogrel, switching from clopidogrel to another P2Y12 inhibitor, or documented completion of the planned DAPT course. Early DAPT discontinuation or switching was defined as discontinuation of either antiplatelet agent or switching from clopidogrel to another P2Y12 inhibitor within 30 days after the index PCI. Reasons for early treatment change were categorized as bleeding or bleeding concern, drug intolerance, physician-guided escalation or switch to another P2Y12 inhibitor, planned surgery or invasive procedure, patient nonadherence, or other documented reasons.

### 2.3 Data collection

Baseline demographic and clinical characteristics were extracted from the electronic medical record at the index admission. Age, sex, and body size (body mass index and body weight) were recorded. Smoking and alcohol consumption were classified as current, former, or never. Comorbidities were defined according to documented physician diagnoses and ongoing treatment, including hypertension, diabetes mellitus, dyslipidemia, chronic kidney disease, prior MI, prior ischemic stroke, and heart failure. Pre-PCI medication history and discharge prescriptions were collected, including long-term antiplatelet or anticoagulant therapy and statin use.

Laboratory measurements were obtained from the first blood test after admission or within 24 h before PCI, including hemoglobin, white blood cell count, platelet count, serum creatinine, and estimated glomerular filtration rate. Alanine aminotransferase, aspartate aminotransferase, low-density lipoprotein cholesterol, high-density lipoprotein cholesterol, triglycerides, and C-reactive protein were recorded from the index hospitalization. Left ventricular ejection fraction was derived from transthoracic echocardiography performed during the index hospitalization.

Procedural and angiographic characteristics were derived from catheterization reports. The index presentation was categorized as stable CAD or ACS (unstable angina, non–ST-segment elevation MI, or ST-segment elevation MI). Coronary anatomy and lesion complexity were documented, including single-*versus* multivessel disease, left main involvement, bifurcation lesions, chronic total occlusion, and severe calcification. Procedural variables included number of implanted stents, total stent length, minimum stent diameter, stent overlap, post-procedural TIMI flow grade, and peri-procedural complications, including coronary dissection and no-reflow. Complex PCI was defined *a priori* as the presence of at least one of the following angiographic or procedural characteristics during the index PCI: left main involvement, multivessel PCI, chronic total occlusion intervention, bifurcation lesion treatment, severe calcification, implantation of three or more stents, total stent length ≥60 mm, or stent overlap. Patients not meeting any of these criteria were classified as having non-complex PCI. Genotype results for CYP2C19, ABCB1, and PON1 were extracted from standardized pharmacogenetic testing reports. For CYP2C19, genotyping results were available for all patients included in the final analytic cohort. Genotyping was performed by the hospital’s clinical pharmacogenetic laboratory using a validated polymerase chain reaction (PCR)-based assay according to standardized laboratory procedures. The tested variants included CYP2C19 *2 and *3, which were categorized as LOF alleles, CYP2C19 *17, which was categorized as an increased-function allele, ABCB1 3435C>T, and PON1 Q192 R. Quality control was performed according to routine laboratory procedures, including internal controls and repeat testing for ambiguous or failed genotype calls. Functional phenotypes were assigned according to the standardized laboratory reports. For the primary analysis, patients classified as intermediate or poor metabolizers because of carriage of at least one CYP2C19 LOF allele were combined into the CYP2C19 LOF phenotype group. Patients without LOF alleles, including those with normal-function or increased-function phenotypes, were categorized as the non-LOF group. ABCB1 3435C>T was categorized as TT, CT, or CC, and PON1 Q192 R was categorized as QQ, QR, or RR according to the laboratory reports.

### 2.4 Endpoint definition and follow-up

The primary endpoint was the occurrence of MACCE after the index PCI. MACCE was defined as a composite of cardiac death, non-fatal MI, ischemic stroke, and TVR, including repeat PCI of the target vessel. Cardiac death was defined as death due to MI, heart failure, malignant arrhythmia, procedure-related cardiac causes, or sudden unexplained death without a clear non-cardiac cause. Non-fatal MI was confirmed by elevated cardiac troponin with clinical, electrocardiographic, imaging, or angiographic evidence of myocardial ischemia. Ischemic stroke was confirmed by a new focal neurological deficit lasting more than 24 h, supported by neurological assessment and brain CT or MRI, with hemorrhagic stroke excluded. TVR was defined as clinically indicated repeat revascularization of the target vessel, documented by coronary angiography or procedural records.

Each suspected endpoint event was adjudicated using prespecified criteria and source documentation, including inpatient and outpatient records, cardiac biomarkers, electrocardiograms, imaging reports, coronary angiography/intervention records, neurological records, and death certificates. For events occurring outside the index institution, external medical records, diagnostic reports, imaging results, angiography/intervention reports, or death certificates were obtained and reviewed whenever available. Telephone-reported events were not counted as MACCE unless supported by medical documentation. Endpoint events were independently reviewed by two investigators blinded to CYP2C19 functional phenotype, and disagreements were resolved by consensus with a senior cardiologist. Time-to-event was calculated from the date of the index PCI to the date of the first qualifying endpoint event; patients without events were censored at the end of follow-up.

### 2.5 Statistical analysis

All statistical analyses were performed using IBM SPSS Statistics, version 28.0 (IBM Corp., Armonk, NY, United States). Continuous variables are presented as mean ± standard deviation or median (interquartile range), and categorical variables as n (%). Between-group comparisons were conducted using the independent-samples t-test (or Welch t-test when appropriate), the Mann–Whitney U test, and the Pearson chi-square test (or Fisher’s exact test when appropriate). Kaplan-Meier methods and the log-rank test were used to compare MACCE -free survival across strata. Risk factors were evaluated using univariable and multivariable logistic regression and Cox proportional hazards regression, reporting odds ratios or hazard ratios with 95% confidence intervals. The proportional hazards assumption was assessed using Schoenfeld residuals. Subgroup analyses were performed by ACS vs. stable CAD, complex PCI vs. non-complex PCI, and CYP2C19 LOF carriers vs. non-carriers, and effect modification was examined using multiplicative interaction terms (e.g., LOF × ACS and LOF × complex PCI) within the Cox framework. Sensitivity analyses included repeating the primary models after excluding patients with early DAPT discontinuation/switching and assessing robustness under the prespecified approach that lost-to-follow-up patients were censored at the last contact date. Events per variable were calculated as the number of observed MACCE events divided by the number of predictor parameters included in each final multivariable model. All tests were two-sided, and P < 0.05 was considered statistically significant.

## 3 Results

### 3.1 Study population

**FIGURE 1:**
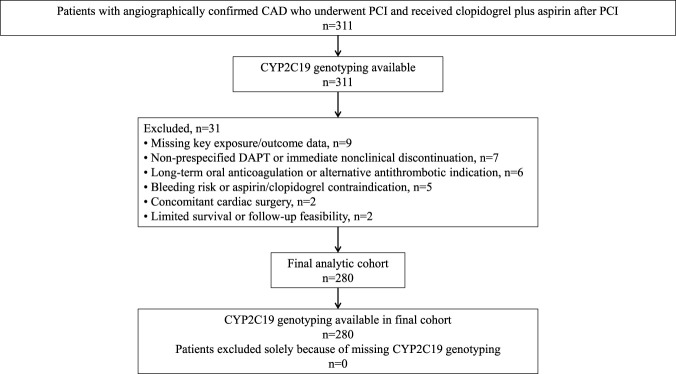
Patient flow diagram.

The patient selection process is shown in Figure 1. During the study period, 311 patients with angiographically confirmed CAD who underwent PCI and were prescribed post-procedural DAPT with clopidogrel plus aspirin were screened for eligibility. At our center, CYP2C19 pharmacogenetic testing was routinely implemented for patients receiving clopidogrel-based DAPT after PCI during the study period. CYP2C19 genotyping results were available for all 311 screened patients, and no otherwise eligible patient was excluded solely because of missing CYP2C19 genotyping. After applying the predefined exclusion criteria, 280 patients were included in the final analytic cohort, all of whom had available CYP2C19 genotyping results.

Follow-up was conducted through outpatient visits, subsequent hospitalization records, and structured telephone interviews, with the follow-up end date defined as the last documented contact on or before December 2025. Follow-up was completed in 272/280 patients (97.1%), and 8/280 patients (2.9%) were lost to follow-up. The median follow-up duration was 32.0 months (interquartile range, 20.0–46.0), and the mean follow-up duration was 33.7 ± 14.2 months. For patients without events, observations were censored at the date of the last confirmed contact.

### 3.2 DAPT duration, adherence, and treatment changes

DAPT exposure and treatment stability are summarized in Supplementary Table S1. In the final analytic cohort, 268 of 280 patients (95.7%) remained on the prespecified clopidogrel–aspirin regimen without early discontinuation or switching during the first 30 days after PCI, whereas 12 patients (4.3%) had early DAPT discontinuation or switching. Among these 12 patients, five experienced MACCE during follow-up. The documented reasons for early treatment change included bleeding or bleeding concern, physician-guided switching to another P2Y12 inhibitor, drug intolerance, planned surgery or invasive procedures, and patient nonadherence. The primary Cox model was repeated after excluding these patients as a sensitivity analysis.

### 3.3 Primary endpoint incidence

In the final analytic cohort of 280 post-PCI patients treated with clopidogrel combined with aspirin, 52 patients experienced at least one MACCE during follow-up, yielding an overall MACCE incidence of 18.6%. Among these first events, cardiac death occurred in eight patients (2.9%), non-fatal MI occurred in 16 patients (5.7%), and ischemic stroke occurred in nine patients (3.2%). TVR, including repeat PCI of the target vessel, was the most frequent component, occurring in 19 patients (6.8%). Each patient contributed only the first qualifying event to the composite endpoint for time-to-event analyses.

### 3.4 Baseline characteristics by MACCE status

Patients with MACCE were older than those without MACCE and had higher prevalences of diabetes mellitus, chronic kidney disease, prior MI, prior ischemic stroke, and heart failure. Pre-PCI medication history, including long-term antiplatelet therapy, anticoagulant use, and statin therapy, was comparable between groups. Compared with patients without MACCE, patients with MACCE had lower hemoglobin, higher serum creatinine, lower eGFR, lower HDL-C, and lower left ventricular ejection fraction. Other lipid parameters and liver enzyme indices did not differ significantly between groups. Several angiographic and procedural characteristics differed by MACCE status. Left main involvement, chronic total occlusion, greater stent number, longer total stent length, smaller minimum stent diameter, stent overlap, post-procedural TIMI flow <3, peri-procedural dissection, and no-reflow were more frequently observed among patients with MACCE. The distribution of index clinical presentation categories did not differ significantly. CYP2C19 functional phenotype distribution differed between groups, with a higher proportion of LOF phenotypes among patients with MACCE, whereas ABCB1 3435C>T and PON1 Q192 R genotype distributions were similar between groups (Table 1).

**TABLE 1:** Recalculated baseline characteristics by MACCE status.

Variable	MACCE (n = 52)	No MACCE (n = 228)	Test statistic	P Value
Age, years	67.20 ± 9.80	62.10 ± 10.70	t = 3.33	0.001
Male sex, n (%)	36 (69.2)	150 (65.8)	χ^2^ = 0.22	0.635
Body mass index, kg/m^2^	25.10 ± 3.40	24.30 ± 3.20	t = 1.55	0.126
Weight, kg	71.40 ± 11.80	69.20 ± 12.10	t = 1.21	0.231
Smoking status, n (%)	Current 20 (38.5); former 12 (23.1); never 20 (38.5)	Current 60 (26.3); former 48 (21.1); never 120 (52.6)	χ^2^ = 3.97 (df = 2)	0.138
Alcohol consumption, n (%)	Current 10 (19.2); former 8 (15.4); never 34 (65.4)	Current 30 (13.2); former 40 (17.5); never 158 (69.3)	χ^2^ = 1.30 (df = 2)	0.521
Hypertension, n (%)	36 (69.2)	130 (57.0)	χ^2^ = 2.62	0.106
Diabetes mellitus, n (%)	22 (42.3)	54 (23.7)	χ^2^ = 7.43	0.006
Dyslipidemia, n (%)	30 (57.7)	108 (47.4)	χ^2^ = 1.81	0.179
Chronic kidney disease, n (%)	14 (26.9)	22 (9.6)	χ^2^ = 11.28	<0.001
Prior MI, n (%)	16 (30.8)	38 (16.7)	χ^2^ = 5.41	0.020
Prior ischemic stroke, n (%)	9 (17.3)	18 (7.9)	χ^2^ = 4.31	0.038
Heart failure, n (%)	12 (23.1)	20 (8.8)	χ^2^ = 8.56	0.003
Pre-PCI long-term antiplatelet therapy, n (%)	18 (34.6)	70 (30.7)	χ^2^ = 0.30	0.583
Pre-PCI anticoagulant use, n (%)	3 (5.8)	10 (4.4)	χ^2^ = 0.18	0.669
Pre-PCI statin therapy, n (%)	32 (61.5)	150 (65.8)	χ^2^ = 0.34	0.562
Hemoglobin, g/L	126.00 ± 16.00	133.00 ± 15.00	t = −2.88	0.005
White blood cell count, ×10^9^/L	7.60 ± 2.10	7.10 ± 1.90	t = 1.58	0.119
Platelet count, ×10^9^/L	210.00 ± 55.00	222.00 ± 58.00	t = −1.41	0.164
Serum creatinine, µmol/L	98.00 ± 28.00	84.00 ± 22.00	t = 3.38	0.001
eGFR, ml/min/1.73 m^2^	68.00 ± 18.00	82.00 ± 16.00	t = −5.16	<0.001
ALT, u/l	24.00 ± 11.00	26.00 ± 12.00	t = −1.16	0.248
AST, u/l	22.00 ± 9.00	23.00 ± 10.00	t = −0.71	0.481
LDL-C, mmol/L	2.42 ± 0.78	2.28 ± 0.74	t = 1.18	0.242
HDL-C, mmol/L	1.00 ± 0.22	1.07 ± 0.25	t = −2.02	0.047
Triglycerides, mmol/L	1.78 ± 0.92	1.62 ± 0.88	t = 1.14	0.258
C-reactive protein, mg/L (median [IQR])	3.6 [1.8–7.4]	2.4 [1.2–5.1]	U = 7,808; Z = 3.57*	<0.001*
Left ventricular ejection fraction, %	52.40 ± 8.60	56.80 ± 7.90	t = −3.38	0.001
Index presentation, n (%)	Stable CAD 18 (34.6); UA 16 (30.8); NSTEMI 10 (19.2); STEMI 8 (15.4)	Stable CAD 120 (52.6); UA 60 (26.3); NSTEMI 30 (13.2); STEMI 18 (7.9)	χ^2^ = 6.75 (df = 3)	0.080
Multivessel disease, n (%)	34 (65.4)	116 (50.9)	χ^2^ = 3.58	0.058
Left main involvement, n (%)	8 (15.4)	14 (6.1)	χ^2^ = 5.00	0.025
Bifurcation lesion, n (%)	18 (34.6)	52 (22.8)	χ^2^ = 3.15	0.076
Chronic total occlusion, n (%)	10 (19.2)	20 (8.8)	χ^2^ = 4.84	0.028
Severe calcification, n (%)	15 (28.8)	40 (17.5)	χ^2^ = 3.43	0.064
Number of implanted stents	2.30 ± 1.00	1.80 ± 0.90	t = 3.31	0.001
Total stent length, mm	48.00 ± 22.00	36.00 ± 18.00	t = 3.66	<0.001
Minimum stent diameter, mm	2.75 ± 0.32	2.85 ± 0.34	t = −2.01	0.048
Stent overlap, n (%)	14 (26.9)	32 (14.0)	χ^2^ = 5.12	0.024
Post-procedural TIMI flow <3, n (%)	6 (11.5)	8 (3.5)	χ^2^ = 5.75	0.017
Peri-procedural dissection, n (%)	4 (7.7)	5 (2.2)	χ^2^ = 4.12	0.042
No-reflow, n (%)	5 (9.6)	6 (2.6)	χ^2^ = 5.47	0.019
CYP2C19 functional phenotype, n (%)	LOF 32 (61.5); normal 16 (30.8); increased 4 (7.7)	LOF 88 (38.6); normal 120 (52.6); increased 20 (8.8)	χ^2^ = 9.42 (df = 2)	0.009
ABCB1 3435C>T, n (%)	TT 18 (34.6); CT 24 (46.2); CC 10 (19.2)	TT 60 (26.3); CT 110 (48.2); CC 58 (25.4)	χ^2^ = 1.76 (df = 2)	0.415
PON1 Q192R, n (%)	QQ 20 (38.5); QR 24 (46.2); RR 8 (15.4)	QQ 110 (48.2); QR 94 (41.2); RR 24 (10.5)	χ^2^ = 1.99 (df = 2)	0.369

Abbreviations: MACCE, major adverse cardiac and cerebrovascular events; PCI, percutaneous coronary intervention; BMI, body mass index; MI, myocardial infarction; eGFR, estimated glomerular filtration rate; ALT, alanine aminotransferase; AST, aspartate aminotransferase; LDL-C, low-density lipoprotein cholesterol; HDL-C, high-density lipoprotein cholesterol; CRP, C-reactive protein; IQR, interquartile range; LVEF, left ventricular ejection fraction; CAD, coronary artery disease; ACS, acute coronary syndrome; UA, unstable angina; NSTEMI, non-ST-segment elevation myocardial infarction; STEMI, ST-segment elevation myocardial infarction; TIMI, thrombolysis in myocardial infarction; CYP2C19, cytochrome P450 family two subfamily C member 19; LOF, loss-of-function; ABCB1, ATP-binding cassette subfamily B member 1; PON1, paraoxonase 1.

### 3.5 Univariable analysis of risk factors for MACCE

Univariable logistic regression was performed to evaluate candidate variables associated with MACCE during follow-up (Table 2). Older age was associated with MACCE. Among clinical comorbidities, diabetes mellitus, chronic kidney disease, prior MI, prior ischemic stroke, and heart failure showed statistically significant associations with MACCE. Hemoglobin, serum creatinine, eGFR, HDL-C, C-reactive protein, and left ventricular ejection fraction were also associated with MACCE in univariable analysis. Index ACS presentation and several angiographic or procedural variables, including left main involvement, chronic total occlusion, number of implanted stents, total stent length, stent overlap, post-procedural TIMI flow <3, and no-reflow, were associated with MACCE. In the pharmacogenetic analysis, CYP2C19 LOF phenotype was associated with MACCE, whereas ABCB1 3435 TT genotype and PON1 192 RR genotype were not. Variables with P < 0.10 were selected for multivariable modeling, and clinically relevant covariates were retained irrespective of univariable significance.

**TABLE 2:** Univariable logistic regression for MACCE.

Candidate factor	OR	95% CI	P Value
Age (per 10 years)	1.62	1.23–2.14	0.001
Male sex (yes vs. no)	1.16	0.61–2.22	0.651
BMI (per 1 kg/m^2^)	1.06	0.98–1.15	0.141
Current smoking (yes vs. no)	1.74	0.96–3.16	0.069
Current alcohol use (yes vs. no)	1.56	0.74–3.27	0.240
Hypertension (yes vs. no)	1.70	0.88–3.29	0.112
Diabetes mellitus (yes vs. no)	2.38	1.26–4.50	0.008
Dyslipidemia (yes vs. no)	1.52	0.82–2.81	0.185
Chronic kidney disease (yes vs. no)	3.47	1.66–7.24	0.001
Prior MI (yes vs. no)	2.22	1.12–4.38	0.023
Prior ischemic stroke (yes vs. no)	2.44	1.03–5.77	0.043
Heart failure (yes vs. no)	3.12	1.42–6.86	0.005
Pre-PCI long-term antiplatelet therapy (yes vs. no)	1.20	0.64–2.25	0.569
Hemoglobin (per 10 g/L)	0.74	0.60–0.91	0.005
Creatinine (per 10 μmol/L)	1.18	1.08–1.29	<0.001
eGFR (per 10 mL/min/1.73 m^2^)	0.62	0.52–0.74	<0.001
HDL-C (per 0.1 mmol/L)	0.88	0.78–0.99	0.032
CRP (per 1 mg/L)	1.07	1.03–1.11	0.001
LVEF (per 5%)	0.74	0.62–0.88	0.001
ACS at index (yes vs. no)	2.10	1.14–3.88	0.018
Multivessel disease (yes vs. no)	1.82	0.98–3.37	0.059
Left main involvement (yes vs. no)	2.78	1.10–7.02	0.031
Chronic total occlusion (yes vs. no)	2.48	1.07–5.75	0.034
Number of implanted stents (per 1 stent)	1.58	1.18–2.13	0.002
Total stent length (per 10 mm)	1.28	1.13–1.45	<0.001
Stent overlap (yes vs. no)	2.26	1.12–4.55	0.023
Post-procedural TIMI flow <3 (yes vs. no)	3.61	1.20–10.86	0.022
No-reflow (yes vs. no)	3.95	1.23–12.66	0.021
CYP2C19 loss-of-function phenotype (yes vs. no)	2.54	1.38–4.69	0.003
ABCB1 3435 TT genotype (yes vs. non-TT)	1.49	0.79–2.82	0.214
PON1 192 R R genotype (yes vs. non-RR)	1.56	0.69–3.50	0.287

Abbreviations: OR, odds ratio; CI, confidence interval; MACCE, major adverse cardiac and cerebrovascular events; BMI, body mass index; PCI, percutaneous coronary intervention; MI, myocardial infarction; eGFR, estimated glomerular filtration rate; HDL-C, high-density lipoprotein cholesterol; CRP, C-reactive protein; LVEF, left ventricular ejection fraction; ACS, acute coronary syndrome; TIMI, thrombolysis in myocardial infarction; CYP2C19, cytochrome P450 family two subfamily C member 19; LOF, loss-of-function; ABCB1, ATP-binding cassette subfamily B member 1; PON1, paraoxonase 1.

### 3.6 Primary multivariable Cox proportional hazards analysis

Given the time-to-event nature of the primary endpoint, the multivariable Cox proportional hazards model was considered the primary analysis for identifying independent predictors of time to first MACCE after PCI. The primary multivariable Cox model included seven predictor parameters for 52 MACCE events, corresponding to approximately 7.4 events per predictor parameter. After multivariable adjustment, age, diabetes mellitus, eGFR, LVEF, total stent length, post-procedural TIMI flow <3, and CYP2C19 LOF phenotype remained statistically associated with time to first MACCE. Older age and diabetes mellitus were associated with a higher hazard of MACCE, whereas higher eGFR and higher LVEF were associated with a lower hazard. Greater total stent length and post-procedural TIMI flow <3 were also associated with a higher hazard of MACCE. In the pharmacogenetic analysis, CYP2C19 LOF phenotype remained associated with time to first MACCE after adjustment for clinical and procedural covariates. The proportional hazards assumption was evaluated using Schoenfeld residuals. No covariate showed evidence of non-proportionality, and the global test supported the proportional hazards assumption (Table 3).

**TABLE 3:** Primary multivariable Cox proportional hazards model and proportional hazards assumption tests for time to first MACCE.

Predictor	Adjusted HR	95% CI	P Value	Schoenfeld PH test P value
Age (per 10 years)	1.36	1.07–1.73	0.012	0.410
Diabetes mellitus (yes vs. no)	1.62	1.01–2.61	0.045	0.290
eGFR (per 10 mL/min/1.73 m^2^)	0.79	0.68–0.92	0.002	0.180
LVEF (per 5%)	0.84	0.73–0.97	0.018	0.330
Total stent length (per 10 mm)	1.15	1.05–1.26	0.003	0.560
Post-procedural TIMI flow <3 (yes vs. no)	2.28	1.09–4.76	0.028	0.220
CYP2C19 loss-of-function phenotype (yes vs. no)	1.74	1.10–2.75	0.018	0.470
Global PH test	—	—	—	0.640

Abbreviations: MACCE, major adverse cardiac and cerebrovascular events; HR, hazard ratio; CI, confidence interval; PH, proportional hazards; eGFR, estimated glomerular filtration rate; LVEF, left ventricular ejection fraction; TIMI, thrombolysis in myocardial infarction; CYP2C19, cytochrome P450 family two subfamily C member 19; LOF, loss-of-function.

### 3.7 Kaplan-Meier analysis stratified by CYP2C19 LOF status

**FIGURE 2:**
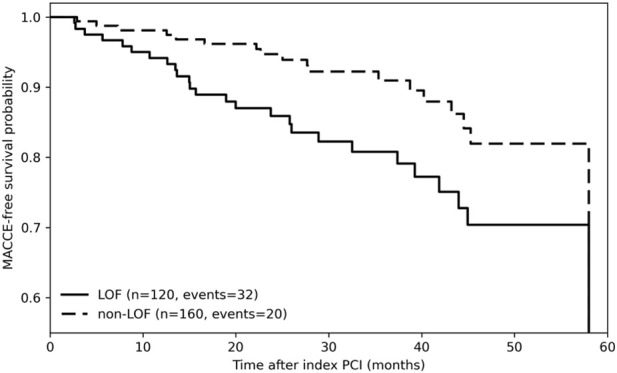
Kaplan–Meier curves for MACCE-free survival stratified by CYP2C19 LOF status. Kaplan–Meier curves showing MACCE-free survival after the index PCI according to CYP2C19 loss-of-function phenotype. The solid line represents patients with the CYP2C19 LOF phenotype, and the dashed line represents patients without the CYP2C19 LOF phenotype. Patients with the CYP2C19 LOF phenotype showed lower MACCE-free survival than non-LOF patients. MACCE, major adverse cardiac and cerebrovascular events; PCI, percutaneous coronary intervention; LOF, loss-of-function.

Kaplan-Meier analysis showed that patients with the CYP2C19 LOF phenotype had lower MACCE-free survival than non-LOF patients during follow-up (Figure 2; Supplementary Table S2). MACCE occurred in 32 of 120 patients in the LOF group and in 20 of 160 patients in the non-LOF group. The estimated MACCE-free survival rates at 12, 24, 36, and 48 months were 94.2%, 85.9%, 80.8%, and 70.4% in the LOF group, respectively, compared with 98.1%, 94.7%, 91.0%, and 82.0% in the non-LOF group. The difference between groups was statistically significant by the log-rank test (P = 0.007), supporting the time-to-event findings from the primary Cox regression analysis.

### 3.8 Secondary multivariable logistic regression analysis

Multivariable logistic regression was performed as a secondary supportive analysis to evaluate factors associated with MACCE occurrence during follow-up. The secondary multivariable logistic regression model included eight predictor parameters for 52 MACCE events, corresponding to approximately 6.5 events per predictor parameter. In this secondary model, age, diabetes mellitus, eGFR, LVEF, total stent length, post-procedural TIMI flow <3, and CYP2C19 LOF phenotype remained statistically associated with MACCE. Older age, diabetes mellitus, greater total stent length, post-procedural TIMI flow <3, and CYP2C19 LOF phenotype were associated with higher odds of MACCE, whereas higher eGFR and higher LVEF were associated with lower odds. Index ACS presentation did not reach statistical significance after adjustment (Table 4).

**TABLE 4:** Secondary multivariable logistic regression for MACCE.

Predictor	Adjusted OR	95% CI	P Value
Age (per 10 years)	1.43	1.05–1.95	0.024
Diabetes mellitus (yes vs. no)	1.92	1.01–3.66	0.046
eGFR (per 10 mL/min/1.73 m^2^)	0.72	0.59–0.88	0.001
LVEF (per 5%)	0.80	0.66–0.96	0.016
ACS at index (yes vs. no)	1.71	0.91–3.22	0.095
Total stent length (per 10 mm)	1.19	1.05–1.36	0.007
Post-procedural TIMI flow <3 (yes vs. no)	2.86	1.02–8.04	0.046
CYP2C19 loss-of-function phenotype (yes vs. no)	2.06	1.08–3.93	0.028

Abbreviations: OR, odds ratio; CI, confidence interval; MACCE, major adverse cardiac and cerebrovascular events; eGFR, estimated glomerular filtration rate; LVEF, left ventricular ejection fraction; ACS, acute coronary syndrome; TIMI, thrombolysis in myocardial infarction; CYP2C19, cytochrome P450 family two subfamily C member 19; LOF, loss-of-function.

### 3.9 Subgroup and interaction analyses

In stratified Cox analyses, the association between CYP2C19 LOF status and time to first MACCE differed by clinical presentation and procedural complexity. CYP2C19 LOF status was statistically associated with MACCE among patients with ACS, but not among patients with stable CAD, with a significant LOF × ACS interaction. Similarly, CYP2C19 LOF status was statistically associated with MACCE in the complex PCI subgroup, but not in the non-complex PCI subgroup, with a significant LOF × complex PCI interaction. These findings indicate subgroup heterogeneity in the association between CYP2C19 LOF status and MACCE (Table 5).

**TABLE 5:** Subgroup-specific association of CYP2C19 LOF with time to first MACCE and interaction testing.

Stratification	Subgroup (n)	Events (n)	Adjusted HR for LOF vs. non-LOF	95% CI	P Value	Interaction P value
Clinical presentation	ACS (142)	34	2.15	1.26–3.68	0.005	0.041 (LOF × ACS)
	Stable CAD (138)	18	1.08	0.52–2.23	0.838	​
Procedural complexity	Complex PCI (88)	26	2.48	1.33–4.62	0.004	0.032 (LOF × Complex PCI)
	Non-complex PCI (192)	26	1.29	0.70–2.36	0.417	​

Abbreviations: ACS, acute coronary syndrome; CAD, coronary artery disease; PCI, percutaneous coronary intervention; MACCE, major adverse cardiac and cerebrovascular events; HR, hazard ratio; CI, confidence interval; CYP2C19, cytochrome P450 family two subfamily C member 19; LOF, loss-of-function.

### 3.10 Sensitivity analyses and assessment of missing data and loss to follow-up

Sensitivity analyses were performed to assess the influence of treatment changes, incomplete follow-up, and missing covariate data on the primary time-to-event findings. These analyses included: (1) exclusion of patients with early discontinuation or switching of DAPT within 30 days after the index PCI; (2) exclusion of patients lost to follow-up; and (3) myocardial infarction (MI) for missing covariates, with pooled estimates generated using Rubin’s rules. No MACCE was documented among the eight patients lost to follow-up before their last confirmed contact. Across the sensitivity analyses, the estimated associations for age, eGFR, LVEF, total stent length, post-procedural TIMI flow <3, and CYP2C19 LOF phenotype remained in the same direction as in the primary Cox model. After excluding patients with early DAPT discontinuation or switching, diabetes mellitus no longer reached statistical significance, whereas the other key predictors remained statistically significant. In the analyses excluding patients lost to follow-up and using MI for missing covariates, the main findings were materially unchanged compared with the primary analysis (Supplementary Table S3).

## 4 Discussion

The present study extends existing evidence on CYP2C19 LOF status in clopidogrel-treated PCI patients by integrating pharmacogenetic information with clinical risk factors and procedural characteristics in a time-to-event framework. Rather than simply confirming the known association between CYP2C19 LOF and adverse outcomes, this study highlights the incremental value of a combined clinical–procedural–genetic assessment for post-PCI risk stratification. In particular, the findings show that CYP2C19 LOF status remained associated with time to first MACCE after adjustment for established clinical and procedural predictors, including age, diabetes mellitus, renal function, left ventricular function, total stent length, and post-procedural TIMI flow. Moreover, the subgroup and interaction analyses suggest that the prognostic relevance of CYP2C19 LOF may be more evident in higher ischemic-risk settings, especially among patients presenting with ACS or undergoing complex PCI. These findings support a more individualized interpretation of CYP2C19 genotyping, in which genetic susceptibility is considered together with baseline clinical vulnerability and procedural complexity when evaluating residual ischemic risk after PCI(Park et al., 2026; Shi et al., 2021).

In this retrospective cohort of CAD patients treated with clopidogrel-based DAPT after PCI, MACCE occurred in 18.6% of patients during a median follow-up of 32.0 months. The composite endpoint included cardiac death, non-fatal MI, ischemic stroke, and TVR, thereby capturing both cardiac and cerebrovascular ischemic outcomes after PCI. The observed event distribution, with TVR as the most frequent component, is clinically plausible in a post-stent population and reflects the multifactorial nature of recurrent ischemic events after coronary intervention. Several conventional clinical factors were associated with MACCE. Older age and diabetes mellitus remained significant in the primary Cox model, consistent with their established roles as markers of systemic atherosclerotic burden and adverse cardiovascular prognosis (Bahrami et al., 2026; Kumar Karn et al., 2025). Renal function and left ventricular systolic function were also associated with time to first MACCE, with lower eGFR and lower LVEF indicating a higher-risk phenotype. These findings emphasize that post-PCI outcomes are not determined solely by antiplatelet responsiveness, but also by baseline organ function, comorbidity burden, and overall cardiovascular reserve (Pitaro et al., 2025; Jayesh et al., 2021). Procedural characteristics also contributed important prognostic information. Greater total stent length and post-procedural TIMI flow <3 remained associated with MACCE in the multivariable Cox model. These findings indicate that lesion complexity, extent of stent implantation, and suboptimal final coronary flow may identify patients with higher residual ischemic risk after PCI. The *a priori* definition of complex PCI further allowed assessment of whether procedural risk modified the association between CYP2C19 LOF and outcomes (Cho et al., 2023; Muramatsu et al., 2023).

The pharmacogenetic findings showed that CYP2C19 LOF phenotype was associated with MACCE in univariable analysis and remained significant in the primary multivariable Cox model. Kaplan-Meier analysis further demonstrated lower MACCE-free survival among CYP2C19 LOF carriers than among non-LOF patients, visually supporting the time-to-event results. In contrast, ABCB1 3435C>T and PON1 Q192R were not associated with MACCE in this cohort. This pattern is consistent with the concept that CYP2C19 is the most clinically actionable genetic determinant of clopidogrel response, whereas the clinical relevance of ABCB1 and PON1 remains less consistent. The overall prevalence of CYP2C19 LOF phenotype in the analytic cohort was 42.9%, whereas the higher proportion observed in the MACCE group reflected enrichment among patients who experienced events. This distribution appears reasonable in the context of East Asian pharmacogenetic data, in which CYP2C19 LOF alleles and intermediate or poor metabolizer phenotypes are relatively common (Prasad et al., 2025; Omar et al., 2025). Therefore, the genotype distribution observed in this cohort is biologically plausible and should be interpreted as population-specific rather than unexpectedly high. Treatment exposure was generally stable during early follow-up, with most patients remaining on the prespecified clopidogrel–aspirin regimen during the first 30 days after PCI. Early DAPT discontinuation or switching occurred in a small proportion of patients, and sensitivity analysis excluding these patients showed results broadly consistent with the primary Cox analysis. Additional sensitivity analyses excluding patients lost to follow-up and using multiple imputation for missing covariates also yielded materially similar findings. These analyses support the robustness of the main results, although they cannot eliminate the possibility of residual confounding inherent to the retrospective observational design (Pereira et al., 2024; van den Broek et al., 2024; Biswas et al., 2025). Overall, the findings indicate that MACCE after PCI in clopidogrel-treated patients was associated with a combination of clinical vulnerability, procedural complexity, reperfusion quality, and CYP2C19 functional phenotype. Importantly, the subgroup and interaction findings for ACS and complex PCI should be interpreted cautiously because of the modest number of events within subgroups and the potential for false-positive interaction results. These analyses should therefore be regarded as hypothesis-generating rather than practice-changing. The results do not establish causality or directly compare alternative P2Y12 inhibitor strategies, but they suggest that the clinical relevance of CYP2C19 LOF status may warrant further evaluation within broader clinical and procedural risk contexts (Rao et al., 2025).

The distribution of CYP2C19 LOF phenotype in this cohort should be interpreted in the context of known East Asian pharmacogenetic patterns. Overall, 120 of 280 patients (42.9%) had the CYP2C19 LOF phenotype, whereas the higher proportion observed in the MACCE group reflected enrichment among patients who developed events. CYP2C19 LOF alleles are known to be more frequent in East Asian populations than in many Western populations (Nieh and Roman, 2025). In an East Asian PCI cohort receiving clopidogrel, Chen et al. reported that 44.9% of patients were intermediate metabolizers and 15.7% were poor metabolizers (Chen et al., 2022). Similarly, a systematic review and meta-analysis by Hardi et al. showed that CYP2C19 variation is common in Southeast Asian populations and is broadly similar to East Asian distributions, with intermediate and poor metabolizer phenotypes associated with increased major adverse cardiovascular events (MACE), hypoaggregation, and clopidogrel resistance among clopidogrel users (Hardi et al., 2025). Therefore, the overall CYP2C19 LOF prevalence in our cohort appears biologically and epidemiologically plausible for an East Asian PCI population treated with clopidogrel, while the higher proportion in the MACCE group supports the observed phenotype–outcome association.

From an implementation perspective, these findings provide several practical considerations for post-PCI management in patients prescribed clopidogrel and aspirin. Patients with older age, diabetes mellitus, impaired renal function, or reduced LVEF may represent a higher-risk clinical phenotype and may require closer surveillance, optimization of secondary prevention, and systematic assessment of adherence. Procedural indicators, including greater total stent length and impaired final TIMI flow, may further help identify patients requiring careful post-procedural evaluation and follow-up planning. In addition, the association between CYP2C19 LOF phenotype and MACCE, particularly among patients with ACS or complex PCI, suggests that CYP2C19 testing may provide clinically relevant information when considered together with clinical and procedural risk factors. In selected high ischemic-risk patients, these findings may inform guideline-concordant discussions regarding antiplatelet optimization, including consideration of alternative P2Y12 inhibition where clinically appropriate (Pereira et al., 2020; Akkaif et al., 2025).

Several limitations should be considered. First, this retrospective observational study remains subject to residual confounding despite multivariable adjustment. Unmeasured factors, such as socioeconomic status, detailed medication adherence, lesion morphology, and operator-related procedural factors, may have influenced the observed associations. In addition, although a parsimonious model was used, the modest number of MACCE events may still introduce some risk of model overfitting. Therefore, the findings should not be interpreted as causal. Second, the cohort was limited to patients receiving clopidogrel plus aspirin after PCI. This allowed focused evaluation of CYP2C19 functional phenotype in a clopidogrel-treated population, but may limit generalizability to patients treated with ticagrelor, prasugrel, or antiplatelet monotherapy. Because antiplatelet therapy was not protocol-assigned, genotype results may have influenced subsequent treatment decisions in routine practice. Although sensitivity analysis excluding early DAPT discontinuation or switching showed directionally consistent results, unmeasured treatment-selection bias cannot be excluded. Third, platelet function testing and clopidogrel active metabolite measurements were unavailable, limiting mechanistic interpretation. Finally, subgroup and interaction analyses were based on modest event counts and should be interpreted as hypothesis-generating. Future prospective multicenter studies with larger sample sizes, standardized antiplatelet protocols, and longer follow-up are needed to validate these findings. Further studies should include different P2Y12 inhibitors, incorporate pharmacodynamic or pharmacokinetic assessments when feasible, and evaluate whether integrating CYP2C19 phenotype with clinical and procedural factors improves post-PCI risk stratification and genotype-informed antiplatelet decision-making.

## 5 Conclusion

In CAD patients receiving clopidogrel plus aspirin after PCI, time to first MACCE was independently associated with older age, diabetes mellitus, reduced renal function, lower LVEF, greater stent burden, impaired post-procedural TIMI flow, and CYP2C19 LOF phenotype. The association between CYP2C19 LOF phenotype and MACCE appeared more pronounced among patients with ACS and those undergoing complex PCI. These findings may support integrated clinical–procedural–genetic risk stratification and provide a rationale for considering genotype-informed antiplatelet assessment in selected high-risk patients.

## Data Availability

The raw data supporting the conclusions of this article will be made available by the authors, without undue reservation.
