# Supplementary material for: Risk factors for Post-PCI cardiovascular events in coronary artery disease patients treated with clopidogrel combined with aspirin
Source: Front Pharmacol. 2026 Jun 18;17:1809310. doi: 10.3389/fphar.2026.1809310 (PMC13323228; doi:10.3389/fphar.2026.1809310)
Supplement: Supplementary file 1 [file Table1.docx]

Supplementary Table S1. DAPT duration, adherence, switching, and discontinuation during follow-up.

| Variable | Overall cohort (n=280) | No early DAPT change (n=268) | Early DAPT discontinuation/switching within 30 days (n=12) |
| --- | --- | --- | --- |
| Planned DAPT duration, months, median (IQR) | 12.0 (6.0–12.0) | 12.0 (6.0–12.0) | 12.0 (6.0–12.0) |
| Actual clopidogrel–aspirin DAPT duration, months, median (IQR) | 11.6 (6.0–12.0) | 11.8 (6.2–12.0) | 0.5 (0.3–0.8) |
| Stable clopidogrel–aspirin DAPT during first 30 days, n (%) | 268 (95.7) | 268 (100.0) | 0 (0.0) |
| Early DAPT discontinuation/switching within 30 days, n (%) | 12 (4.3) | 0 (0.0) | 12 (100.0) |
| Time to early DAPT change, days, median (IQR) | — | — | 16 (10–24) |
| Switching from clopidogrel to another P2Y12 inhibitor, n (%) | 5 (1.8) | 0 (0.0) | 5 (41.7) |
| Discontinuation or interruption of aspirin and/or clopidogrel, n (%) | 7 (2.5) | 0 (0.0) | 7 (58.3) |
| Documented reason for early DAPT change, n (%) |  |  |  |
| Bleeding or bleeding concern | 4 (1.4) | 0 (0.0) | 4 (33.3) |
| Physician-guided switch to another P2Y12 inhibitor | 4 (1.4) | 0 (0.0) | 4 (33.3) |
| Drug intolerance | 2 (0.7) | 0 (0.0) | 2 (16.7) |
| Planned surgery or invasive procedure | 1 (0.4) | 0 (0.0) | 1 (8.3) |
| Patient nonadherence | 1 (0.4) | 0 (0.0) | 1 (8.3) |
| MACCE during follow-up, n (%) | 52 (18.6) | 47 (17.5) | 5 (41.7) |

Abbreviations: DAPT, dual antiplatelet therapy; IQR, interquartile range; MACCE, major adverse cardiac and cerebrovascular events; PCI, percutaneous coronary intervention.

Supplementary Table S2. Kaplan–Meier survival estimates and numbers at risk stratified by CYP2C19 LOF status.

| CYP2C19 group | n | MACCE events, n (%) | Censored, n (%) | At risk, 0 mo | At risk, 12 mo | At risk, 24 mo | At risk, 36 mo | At risk, 48 mo | MACCE-free survival at 12 mo | MACCE-free survival at 24 mo | MACCE-free survival at 36 mo | MACCE-free survival at 48 mo |
| --- | --- | --- | --- | --- | --- | --- | --- | --- | --- | --- | --- | --- |
| CYP2C19 LOF | 120 | 32 (26.7) | 88 (73.3) | 120 | 109 | 78 | 49 | 22 | 94.2% | 85.90% | 80.80% | 70.40% |
| CYP2C19 non-LOF | 160 | 20 (12.5) | 140 (87.5) | 160 | 152 | 125 | 67 | 28 | 98.1% | 94.70% | 91.00% | 82.00% |

Abbreviations: CYP2C19, cytochrome P450 family 2 subfamily C member 19; LOF, loss-of-function; MACCE, major adverse cardiac and cerebrovascular events; mo, months.

Supplementary Table S3. Sensitivity analyses for the multivariable Cox model of time to first MACCE.

| Analysis scenario | Sample size (n) | Events (n) | Age (per 10y) HR (95% CI), P | Diabetes HR (95% CI), P | eGFR (per 10) HR (95% CI), P | LVEF (per 5%) HR (95% CI), P | Total stent length (per 10mm) HR (95% CI), P | TIMI<3 HR (95% CI), P | CYP2C19 LOF HR (95% CI), P |
| --- | --- | --- | --- | --- | --- | --- | --- | --- | --- |
| Primary analysis (complete-case Cox) | 280 | 52 | 1.36 (1.07–1.73), 0.012 | 1.62 (1.01–2.61), 0.045 | 0.79 (0.68–0.92), 0.002 | 0.84 (0.73–0.97), 0.018 | 1.15 (1.05–1.26), 0.003 | 2.28 (1.09–4.76), 0.028 | 1.74 (1.10–2.75), 0.018 |
| Exclude early discontinuation/switching of DAPT within 30 days* | 268 | 47 | 1.34 (1.04–1.72), 0.023 | 1.59 (0.96–2.64), 0.071 | 0.80 (0.68–0.94), 0.007 | 0.85 (0.73–0.99), 0.033 | 1.14 (1.03–1.26), 0.010 | 2.20 (1.00–4.85), 0.049 | 1.69 (1.05–2.73), 0.031 |
| Exclude patients lost to follow-up† | 272 | 52 | 1.37 (1.07–1.75), 0.011 | 1.64 (1.01–2.66), 0.044 | 0.78 (0.67–0.91), 0.002 | 0.83 (0.72–0.96), 0.014 | 1.16 (1.05–1.27), 0.002 | 2.31 (1.10–4.86), 0.027 | 1.76 (1.11–2.80), 0.016 |
| Multiple imputation (MI) for missing covariates‡ | 280 | 52 | 1.35 (1.06–1.72), 0.016 | 1.60 (1.00–2.57), 0.049 | 0.80 (0.69–0.93), 0.003 | 0.85 (0.74–0.97), 0.019 | 1.15 (1.05–1.25), 0.002 | 2.24 (1.08–4.66), 0.030 | 1.72 (1.10–2.70), 0.017 |

* Early DAPT change was defined as discontinuation of either antiplatelet agent or switching to an alternative P2Y12 inhibitor within 30 days after the index PCI.

† This sensitivity analysis excluded the 8 patients who were lost to follow-up. No MACCE was documented in these patients before the date of last confirmed contact; therefore, the number of events remained 52 after their exclusion.

‡ MI was performed using multiple imputation by chained equations (m=20 imputations). The imputation model included follow-up time, event indicator, and all candidate covariates; pooled estimates were obtained using Rubin’s rules.

Abbreviations: MACCE, major adverse cardiac and cerebrovascular events; HR, hazard ratio; CI, confidence interval; DAPT, dual antiplatelet therapy; MI, multiple imputation; PCI, percutaneous coronary intervention; eGFR, estimated glomerular filtration rate; LVEF, left ventricular ejection fraction; TIMI, Thrombolysis In Myocardial Infarction; LOF, loss-of-function.
